# Multivariate FMRI Signatures of Learning in a Hebb Repetition Paradigm With Tone Sequences

**DOI:** 10.3389/fneur.2021.674275

**Published:** 2021-11-29

**Authors:** Corey Loo, Andy C. H. Lee, Bradley R. Buchsbaum

**Affiliations:** ^1^Rotman Research Institute, Baycrest, Toronto, ON, Canada; ^2^Department of Psychology, University of Toronto, Toronto, ON, Canada

**Keywords:** working memory, learning, fMRI, representational similarity analysis, auditory memory, sequence memory, Hebb repetition effect

## Abstract

Important information from the environment often arrives to the brain in temporally extended sequences. Language, music, actions, and complex events generally unfold over time. When such informational sequences exceed the limited capacity of working memory, the human brain relies on its ability to accumulate information in long-term memory over several encounters with a complex stimulus. A longstanding question in psychology and neuroscience is whether the neural structures associated with working memory storage—often viewed as capacity limited and temporary—have any builtin ability to store information across longer temporal delays. According to the classic Hebbian dual memory theory, temporally local “activity traces” underlie immediate perception and working memory, whereas “structural traces” undergird long-term learning. Here we examine whether brain structures known to be involved in working maintenance of auditory sequences, such as area Spt, also show evidence of memory persistence across trials. We used representational similarity analysis (RSA) and the Hebb repetition paradigm with supracapacity tonal sequences to test whether repeated sequences have distinguishable multivoxel activity patterns in the auditory-motor networks of the brain. We found that, indeed, area Spt and other nodes of the auditory dorsal stream show multivoxel patterns for tone sequences that become gradually more distinct with repetition during working memory for supracapacity tone-sequences. The findings suggest that the structures are important for working memory are not “blank slates,” wiped clean from moment to moment, but rather encode information in a way can lead to cross-trial persistence.

## Introduction

Humans are frequently faced with the need to detect, briefly store, and act upon a sequence of information encountered in the environment. In the canonical (if now anachronistic) example, one reads outs a number from the telephone book and then must retain it in memory—in *working memory*—as one travels from the phone book to the phone. A question that has long interested psychologists and neuroscientists has been how the brain holds on to new arbitrary sequences of information. Such sequences consist of information patterns for which no long-term memory trace can yet exist, even if such traces may exist for the constituent elements of the sequence. For example, using the example of the phone number, although one may have a lifetime's worth of experience with the numerical digits “0” to “9,” there are a very large number of arbitrary arrangements of such numbers (e.g., 428–7,539) whose sequential combinatorial arrangement has never been encountered.

The psychologist Donald Hebb was particularly interested in the neurophysiological mechanisms (“a dual trace” theory) that underlie both the learning and short-term maintenance of sensory- or sensory-motor sequences of the kind exemplified by the phone-number task. Hebb conceived of two main physiological mechanisms that support short-term maintenance and long-term learning, respectively (1). He proposed that short-term maintenance of information is supported by *activity traces* encoded in distributed *cell assemblies* that could achieve temporary persistence through reverberatory activity that relied on sensory-motor connections and synaptic feedback loops. Hebb proposed that long-term learning was mediated by the repeated co-occurrence of synaptic connectivity among sets of neurons, leading to a gradual strengthening of connections—*a structural trace*—between such sets or assemblies.

Hebb (2) fixed upon a difficulty or puzzle of human long-term learning and short-term memory that was not easily accommodated by his dual trace mechanism. On the one hand, he pointed out that the human brain, due in part to its large numbers of neurons and wide expanses of association regions, at any time is dominated by neural activity (“excess neural activity”) that is not currently relevant to learning a *specific* task. Thus, “any random activity in these excess neurons (the ones not needed for the task being learned) is ‘noise’, which must tend to interfere with learning” [(2), p. 38]. Thus, the very flexibility afforded by a large number of neurons, and the high proportion of those neurons devoted to association processes, makes the human brain not especially well-adapted for fast learning of specific tasks due to the swamping of a small signal with large excess neural activity. Thus, the higher animal, despite its increased capacity for flexible behavior, does not necessarily learn specific tasks faster than the lower animal, for example a wasp that succeeds at one-trial learning (3).

A second puzzle raised by Hebb was that the very “duality” of his trace theory seemed to preclude the short-term learning of novel *recombinations* of highly overlearned items. On the one hand, Hebb's “activity trace” is a purely temporary phenomenon that vanishes once the reverberatory activity ceases. On the other hand, the formation of structural traces through repeated co-occurrence proceeds slowly, lest it be so volatile that regularities of the sensory environment (such as the digits 0–9) could not be stably represented over time. Indeed, his theory seemed to predict that novel sequences of random digits exceeding the finite capacity of the reverberatory system, could not be “learned” at all over a sufficiently short period! To explore this seeming paradox Hebb devised the (now eponymously named) “Hebb repetition paradigm.” In this task, a human subject is asked to repeat back a sequence of nine digits over a series of trials; where unbeknownst to the subject, one of the digit sequences repeats on every third trial. His paradigm attempts to expose a lacuna is his own theory, and therefore constituted a strong test of the “dual trace” theory of short- and long-memory. What Hebb, and many subsequent authors have found (4–7), was that indeed, human subjects are able to accumulate gains in the ability to recall the repeated sequence, compared to the interspersed novel filler sequences.

With this historical overview of the Hebb repetition paradigm in mind, we turn to a brief discussion of cognitive psychological and neuroscientific views of the architectural and neuroanatomical foundations of short-term or working memory maintenance. The Working Memory model of Baddeley (8) and Baddeley and Hitch (9) posits that sensory information can be held for brief intervals in domain dependent buffer systems (i.e., dependent of the type of information, e.g., phonological or visuospatial), and that information stored within such buffers is amenable to “refreshing,” rehearsal, and reactivation directed from higher-order motor control regions. One can see a clear resemblance between such interacting sensory-motor systems of Working Memory and Hebb's reverberatory loops (10). Indeed, the “buffers” of Baddeley's Working Memory may be viewed simply as the storehouses of Hebb's activity traces and are therefore purely temporary mechanisms for guiding behavior in the *just-immediate* future. Cognitive neuroscience investigations into the neuroanatomical locations underlying short-term memory storage have identified areas that show elevated activity during the temporary maintenance of various kinds of informational elements (e.g., words, faces, spatial elements). For example, short-term storage of phonological information has been consistently found to rely on the left lateral temporoparietal area, a finding that has been observed in PET studies (11, 12), fMRI (13, 14), magnetoencephalography (15), electrocorticography (16, 17), and neuropsychological studies of patients with selective verbal short-term memory deficits.

There has been some debate, however, about the precise role of such working memory “storage” and “maintenance” regions in the verbal working memory tasks. For example, some have argued for a classic “buffer” interpretation of delay period activity observed in higher-order association areas generally (18), and in the temporoparietal area in the specific context of phonological short-term memory [e.g., (12, 19, 20)]. A more general argument has, moreover, been made that some sort of purely temporary buffer system is required for a functional working memory system, especially when multiple tokens of the same type must be maintained in short-term memory (21). On the other hand, we have argued (10), that the apparent buffer-related activity observed in temporoparietal cortex reflects the operation of a more general sensory-motor integration system that serves as a mediating node between auditory and motor systems involved in speech production and speech perception. By this view (22–24), phonological working memory emerges from reverberatory interactions between regions supporting the acoustic representations of speech, those supporting articulatory representations of speech, and a system supporting the mediation or mapping between these dual codes. Furthermore, through more precise single-subject fMRI mapping (13, 25) and meta-analyses (24), we have clearly localized the hypothesized auditory-motor interface to the posterior most portion of the left planum temporale, a region we have called Spt (Sylvian-parietal-temporal).

If Spt is involved in sensory-motor integration in the auditory domain and in the learning of new sequential word and sound forms, then it seems likely that the region should not only be important for “temporary maintenance” of novel verbal/auditory stimuli, but also should be important for long-term learning of such forms (23, 26). This implies that Spt should be capable of forming “structural traces” of multielement auditory stimuli that emerge as a function of auditory-motor experience. The formation of these structural traces can be observed using the classical Hebbian repetition paradigm, as demonstrated by Kalm et al. (27). Through the repeated presentation of a letter sequence intermixed with novel sequences, they observed the gradual formation and stabilization of repeated-sequence specific representations in the left hippocampus over successive repetitions. In the present study we use a modified version of the classic Hebb Repetition paradigm using 9-element supraspan tone sequences as working memory stimuli. We choose tones, rather than digits or letters, because they are less susceptible to semantic chunking (e.g., common letter strings, such as CIA, FBI, NBA) and place a high load on auditory-motor integration (25). Furthermore, we were interested in whether Spt and other regions of the language network are important for the learning of non-phonological auditory sequences. We hypothesize that Spt and possibly other regions in the auditory-motor network, should show evidence of repetition effects that are sequence-specific, a finding that would be consistent with the formation of a structural—or at least persistent—trace across trials. On the other hand, if Spt is only needed for temporary binding or storage of auditory sequence stimuli, then we should not find any evidence of such persistence. To test these ideas, we use representational similarity analysis (RSA) to analyze the relationships between multivariate patterns among repeated and non-repeating trials as a way to examine whether coherent patterns emerge for sequences that repeat across trials.

## Methods

### Participants

Twenty-six right-handed young adults (age 18–34; 14 female) with normal or corrected-to-normal vision and no history of neurological or psychological disease were recruited through the Baycrest subject pool, tested and paid for their participation. Informed consent was obtained, and the experimental protocol was approved by the Rotman Research Institute's Ethics Board. Subjects were either native or fluent English speakers and had no contraindications for MRI. Data from three of these participants were removed due to data acquisition errors or excessive head movement (>3 mm framewise displacement).

### Behavioral Procedure

On each trial, participants were presented with two successive sequences of tones (a target sequence and a probe sequence), separated by a variable delay, and were asked to determine if the two sequences were identical (e.g., “match” or “mismatch”). The variable delay interval was used to achieve greater decorrelation in the hemodynamic response evoked by different task phases. Tone sequences were presented over 3 s, and consisted of nine tones, each lasting 200 ms with a 150 ms silent gap between each tone. The target (encoded) tone sequences were generated by choosing tones spanning the frequency range between 300 and 2,600 Hz, using proportional increments of 30%. The sequences were then pseudorandomly shuffled to generate unique tone strings. The probe sequences on each trial were defined in one of two ways: for “match” trials the target and probe sequences were identical; and for mismatch trials, two elements of the target tone sequence were transposed, altering the ordering of the sequence but retaining the set of frequencies. The presentation of the study sequence and probe sequence were separated by a silent delay period (3, 6, or 9 s). Participants responded with a button press during the test phase following the variable delay, by pressing (1) match, (2) non-match on an MRI compatible box using the right index and middle finger, respectively.

Crucially, three unique sequences (S1, S2, and S3) were repeated throughout the task at as study sequences (participants were not informed of this manipulation), intermixed with novel sequences. The same repeating sequence (e.g., S1–S1) was never repeated back to back across the encoding phases of two successive trials. We used three repeating sequences, rather than one as was used in the original Hebb paradigm, with the intention to minimize awareness of the repetition manipulation as it has been demonstrated that multiple sequences can be learned in the classic Hebb repetition paradigm at no cost (28, 29). In all, there were four trial types: (1) repeat match, (2) repeat mismatch, (3) novel match, (4) novel mismatch, across three different levels of delay for a total of 12 conditions. The actual composition of the repeating sequences was not the same for each subject; two versions of the task were created, each with different pseudorandomly generated repeating sequences, and each given to half of the subjects.

Participants completed eight scanning runs consisting of 18 trials each, for a total of 144 trials across all runs (36 repeat match, 36 repeat mismatch, 36 novel match, 36 novel mismatch). Over the course of the experiment, each repeating sequence was presented as a target in 24 trials. Trials were separated by an inter-trial interval that was jittered between 4 and 10 s (average: 7 s).

### MRI Acquisition

Participants were scanned with a 3.0-T Siemens MAGNETOM Trio MRI scanner using a 12-channel head coil system. Functional images were acquired using an Echo-planar imaging (EPI) sequence sensitive to BOLD contrast (22 × 22 cm field of view with a 96 × 96 matrix size, resulting in an in-plane resolution of 2.34 × 2.34 mm for each of 24 3.9 mm axial slices; repetition time = 1.37s; echo time = 30 ms; flip angle = 62 degrees). A high-resolution whole-brain magnetization prepared rapid gradient echo (MP-RAGE) 3-D T1-weighted scan (160 slices of 1 mm thickness, 19.2 × 25.6 cm field of view) was also acquired for anatomical localization.

### MRI Pre-processing

Results included in this manuscript come from preprocessing performed using FMRIPREP version stable (30, 31), a Nipype (32, 33) based tool. Each T1w (T1-weighted) volume was corrected for INU (intensity non-uniformity) using N4BiasFieldCorrection v2.1.0 (34) and skull-stripped using antsBrainExtraction.sh v2.1.0 (using the OASIS template). Brain surfaces were reconstructed using recon-all from FreeSurfer v6.0.1 (35), and the brain mask estimated previously was refined with a custom variation of the method to reconcile ANTs-derived and FreeSurfer-derived segmentations of the cortical gray-matter of Mindboggle (36). Spatial normalization to the ICBM 152 Non-linear Asymmetrical template version 2009c (37) was performed through non-linear registration with the antsRegistration tool of ANTs v2.1.0 (38), using brain-extracted versions of both T1w volume and template. Brain tissue segmentation of cerebrospinal fluid (CSF), white-matter (WM) and gray-matter (GM) was performed on the brain-extracted T1w using fast from FSL v5.0.9 (39). Functional data was slice time corrected using 3dTshift from AFNI v16.2.07 (40) and motion corrected using mcflirt (41). “Fieldmap-less” distortion correction was performed by co-registering the functional image to the same-subject T1w image with intensity inverted (42, 43) constrained with an average fieldmap template (44), implemented with antsRegistration (ANTs). This was followed by co-registration to the corresponding T1w using boundary-based registration (45) with six degrees of freedom, using bbregister (FreeSurfer v6.0.1). Motion correcting transformations, field distortion correcting warp, BOLD-to-T1w transformation and T1w-to-template (MNI) warp were concatenated and applied in a single step using antsApplyTransforms (ANTs v2.1.0) using Lanczos interpolation.

Physiological noise regressors were extracted applying CompCor (46). Principal components were estimated for the two CompCor variants: temporal (tCompCor) and anatomical (aCompCor). A mask to exclude signal with cortical origin was obtained by eroding the brain mask, ensuring it only contained subcortical structures. Six tCompCor components were then calculated including only the top 5% variable voxels within that subcortical mask. For aCompCor, six components were calculated within the intersection of the subcortical mask and the union of CSF and WM masks calculated in T1w space, after their projection to the native space of each functional run. Frame-wise displacement (47) was calculated for each functional run using the implementation of Nipype. Many internal operations of FMRIPREP use Nilearn (48), principally within the BOLD-processing workflow. For more details of the pipeline see https://fmriprep.readthedocs.io/en/stable/workflows.html.

### Regions of Interest

**Figure 1:**
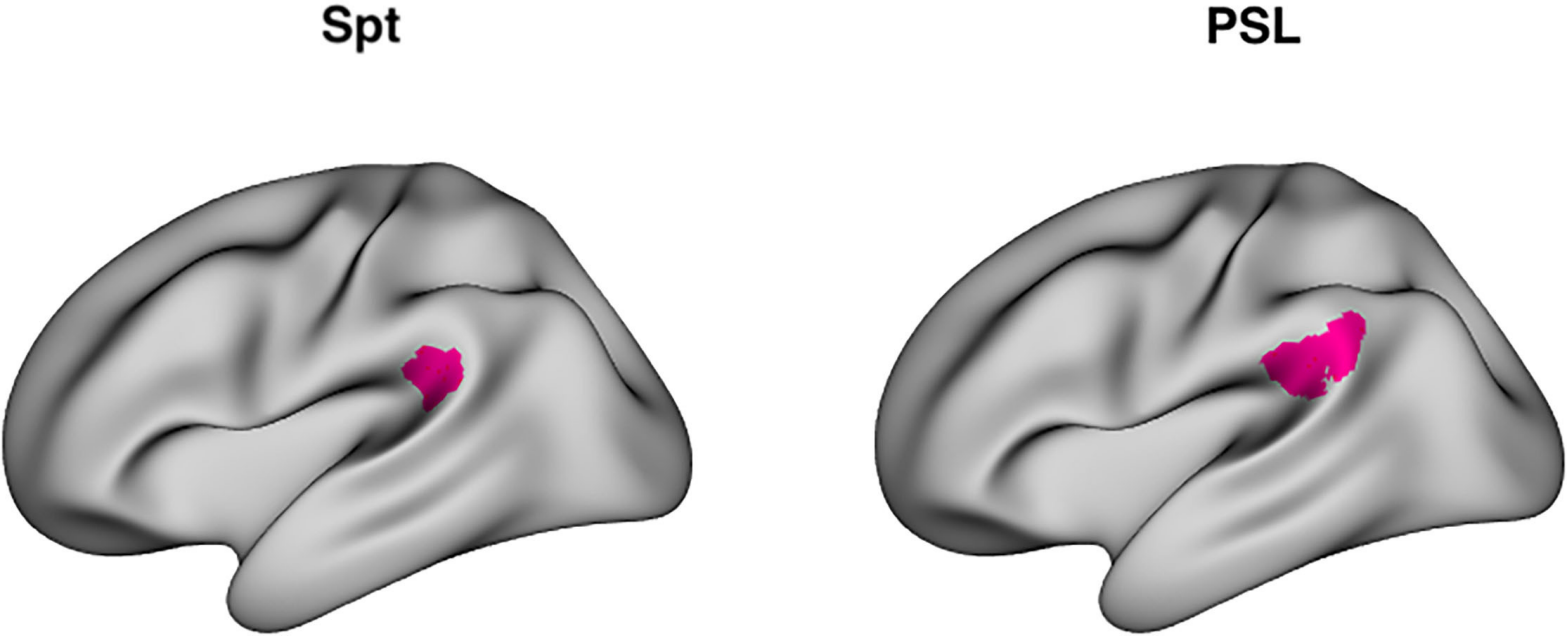
Area Spt and PSL. Left: Lateral view of peak coordinates (−51, −43, 20) of Area Spt as reported in Buchsbaum et al. (24) meta-analysis of auditory-verbal working memory studies. Right: lateral view of PSL (perisylvian language area), a “parcel” fined by a multimodal clustering approach of Glasser et al. (49). The peak coordinates of Spt are contained within the boundaries of PSL [PSL has a right-hemisphere homolog whereas right-sided Spt activity sometimes evident in single subjects (25), but often fails to reach significance in group level analyses].

RSA analysis, the main focus of the present work, examines distributed patterns of activity over a multivoxel ROI. A key question in the current investigation is whether Spt shows evidence of the formation of longer-lasting memory traces as a function of sequence repetition; however, Spt is traditionally defined using a univariate conjunction of auditory perceptual activity and rehearsal or short-term memory related activity as measured during a silent delay period (25). The Glasser parcellation (49) defines a region named Perisylvian Language Area (PSL) that is highly spatially overlapping with area Spt, and localizes to the parieto-temporal boundary at the back of the Sylvian fissure. Moreover, its average MNI coordinates (−57, −41, 24) are very close to the meta-analytically determined peak coordinates of Spt (−51, −43, 20) (24) (Figure 1). Here we use the area PSL of the Glasser atlas as a convenient multivoxel proxy for Spt and which can easily be used to define a consistent set of voxels for RSA analyses without requiring a complex subject-specific search strategy. Using the Glasser parcellation also allows us to easily extend our RSA analysis to other regions in lateral temporal and frontal regions in the broader auditory-motor network, as well as for a fully exploratory analyses in the whole neocortex.

### Univariate Analysis of Voxelwise Activity

In the current study, we used univariate analysis to identify regions of interest to constrain and limit the set of regions that were examined in the RSA analyses. Our goal was to identify areas of the brain that are responsive during tone sequence perception, tone sequence working memory maintenance (e.g., delay period activity), and the conjunction of the two. To that end, we conducted a whole-brain voxelwise univariate analysis in which encoding phase (0–3 s after trial start), delay phase (3 s after trial start), and probe phase (6, 9, or 12 s after trial start, depending on variable delay length) were modeled with an SPM canonical hemodynamic response function convolved with a delta function whose width was equal to the duration of each event type (e.g., encoding: 3 s, delay: 3 s, 6 s, or 9s, probe: 3 s). Separate regressors for encoding, delay, and probe phases were generated for novel and repeat trials; and for the probe phases, novel and repeat trials were further separated depending on whether the probe tone sequence was a “match” or “mismatch.” Low frequency drift and nuisance signals were further modeled with a 5-degree polynomial, and a set of 5 “aCompCorr” (46) components produced by FMRIPREP. For the purposes of identifying regions generally active during tone sequence encoding and short-term maintenance, contrasts were computed to identify regions with above-baseline levels of activity during these encoding and delay phases, collapsing across the experimental manipulation of repetition.

**Figure 2:**
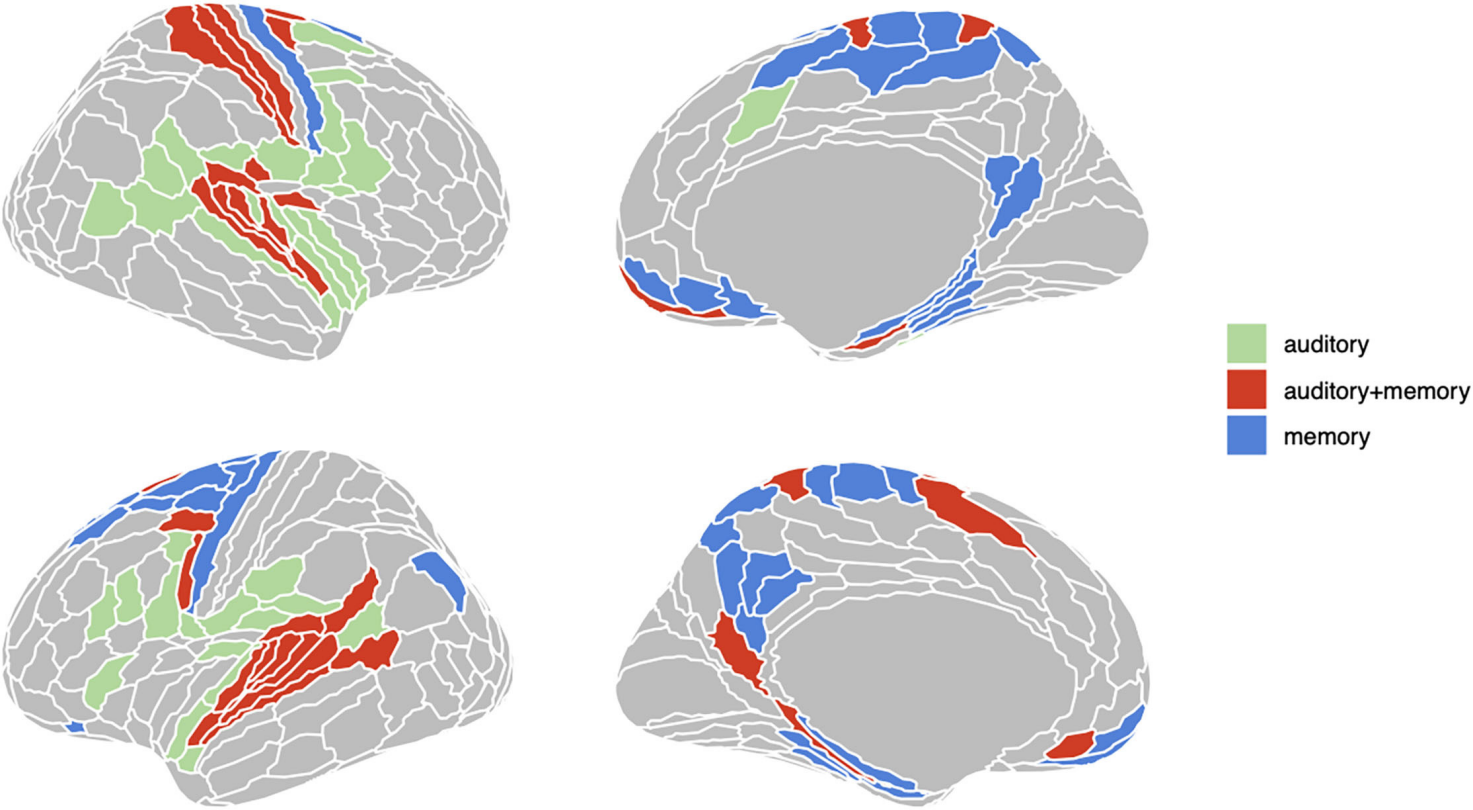
Three groups of ROIs based on univariate analysis. Regions in green are active (FDR < .05) only during encoding phase; regions in red are active both during encoding and delay period; regions in red are only active during delay period.

To define sets of ROIs for RSA analyses we first computed the average beta coefficient across voxels in each of the 360 Glasser ROIs for the encoding and delay phase regressors. We then computed one-sample *t*-test in each ROI for both phases, yielding 360 t-statistics and their associated *p*-values. FDR thresholds (50, 51) for each set of (encoding, delay) 360 *p*-values were then computed using the R function “p.adjust” with FDR < 0.05. FDR provides improved sensitivity relative to Bonferroni correction and flexibly adapts to the observed distribution of *p*-values. Finally, three sets of ROIs were constructed as the following conjunction: “auditory” [*p*(FDR) encoding < 0.05 and *p*(FDR) delay > 0.05], “auditory + memory” [*p*(FDR) encoding < 0.05 and *p*(FDR) delay < 0.05], and “memory” [*p*(FDR) encoding > 0.05 and *p*(FDR) delay < 0.05]. The three sets of ROIs defined in this way are displayed in Figure 2.

### Representational Similarity Analysis of Tone-Sequences

We used RSA analysis (52) to examine whether multivariate patterns of activity within a given ROI were more similar for the *same* repeated tone sequence (during encoding, delay, and probe phases) than among *different* repeated tone sequences. We thus asked whether the set of presentations for repeated sequence S1 showed more similar patterns of activity with one another (e.g., within-sequence similarity) than with the other (e.g., between-sequence similarity) repeated sequences (S2 and S3). In other words, were the set of S1 repeated sequences distinguishable from the set of repeated S2 and S3 sequences?

We constructed a representational dissimilarity matrix (RDM) for the set of repeat trials by computing the pairwise similarity between all repeating tone-sequences using the Earth Mover's Distance (EMD) between tone-sequence pairs. The EMD is a measure of the cost of transforming one tone-sequence into another, considering the order and magnitude of the frequencies (53). Two identical sequences naturally have an EMD = 0, and different sequences have distances which depend on the difference between the distributions of their frequencies over lag. The standard way of describing EMD is in terms of the cost of moving piles of dirt from location to fill up some holes in the ground in another location. In our case it is “costlier” to move tones with different frequencies across larger distances (serial positions).

Because patterns of activity might be driven by tone-sequence similarity even for non-repeated sequences (e.g., novel trials) we also generated an RDM using EMD for the set of novel trials. We used this RDM as a baseline measure of the extent to which similar tone sequences have similar neural representations, even when those sequences are only presented once. By subtracting the “novel RDM” from the “repeat RDM,” we could test whether repeated sequences have more similar activity patterns than novel sequences, even after accounting for inter-item sequence similarity.

To extract estimates of fMRI activity for each phase (encoding, delay, probe) and trial of the task, we implemented a single trial beta estimation procedure using partial least squares (PLS). This involves the following steps: (1) time-series data are first denoised with the same matrix of nuisance covariates described above (5-degree polynomial and 5 CompCorr components); (2) a design matrix, X, was constructed by modeling each event as a separate regressor, yielding one regressor for each trial; a “fixed effect” consisting of the row-wise sum of X was added to the design matrix (addition of this term regularizes the trialwise estimates); (3) a univariate response vector, Y, was specified as the denoised time-series for a given voxel; (4) the PLS model was estimated using the R package [“pls”; (54)] and beta coefficients (i.e., trialwise activity estimates) were extracted. The above process was repeated for each phase (encoding, delay, and probe). These trialwise beta coefficients could then be matched up in a one-to-one relationship with the RDM matrices described above.

## Results

### Behavioral Analysis of Tone-Sequence Recognition Memory

The tone sequence discrimination task was designed to be difficult, as it required the detection of a single transposition of sequentially adjacent tones between two (target and probe) nine-tone sequences. Overall accuracy was at 56.75%, which is above chance performance (*p* = 0.002, one-sample Wilcoxon signed rank test). To examine the effect of delay (3, 6, 9s), probe type (match, mismatch), and sequence type (repeat, novel) we computed a linear mixed effects model with d-prime as the response variable, subject as a random intercept, and random slopes for delay, and sequence type using the R package lme4 (55). There was a main effect of delay [*F*_(2,8.3)_ = 10.8, *p* < 0.0048, Satterthwaite approximation for degrees of freedom] with the best performance at 3s (proportion correct = 0.631; d-prime = 0.698), intermediate performance at 6 s (proportion correct = 0.568; d-prime = 0.356) and worst performance at 9s (proportion correct = 0.504; d-prime = 0.0171). There was no main effect of sequence repetition [*F*_(1,22.67)_ = 0.119, *p* = 0.73], but there was a sequence type vs. delay interaction [*F*_(2,940.8)_ = 5.69, *p* = 0.0035]. Pairwise comparisons across delay conditions, showed that the sequence type by delay interaction was driven by greater d-prime for the 3s delay condition in the novel than in the repeat condition (difference = 0.27, *t* = 2.34, *p* = 0.029) and no differences at other delays (6s: difference beta = −0.19, *t* = −1.572, *p* = 0.12; 9s: difference beta = 0.0147, *t* = *0.1*22, *p* = 0.90).

To test whether performance on repetition trials increased with block number (e.g., better performance on later blocks), we ran a second linear mixed model that incorporated “block number” as a covariate and tested for a block number by sequence repetition interaction, but there was no effect (*p* = 0.45). Thus, performance on repeating sequences was not generally better than performance on novel sequences and this did not change with more repetitions.

### RSA Analyses of Novel and Repeated Sequences

**Figure 3:**
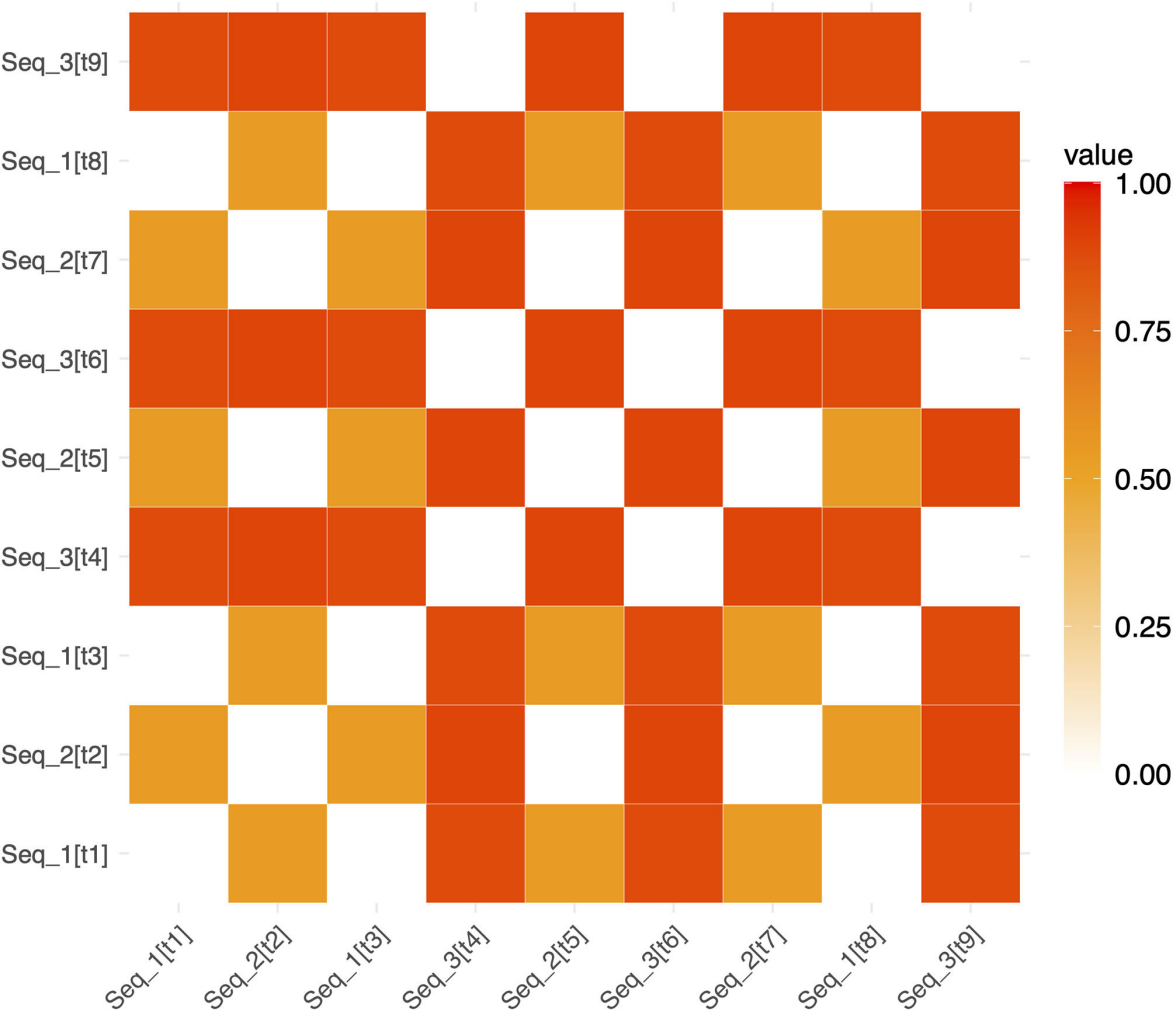
Example representational dissimilarity matrix for repeated sequences. An example RDM for repeated sequences in a single run (for ease of visualization). White squares show pairs of trials involving the identical repeated sequence (and therefore have a dissimilarity of 0). Red and orange squares show the dissimilarity between pairs of non-identical repeating sequences (e.g., Seq1 and Seq2). The notation [tn] refers to the trial number within a run. [t1] refers to “trial 1” and [t2] refers to “trial 2”, etc. (e.g. Seq_2[t7] denotes that Seq2 was presented on trial 7).

**Figure 4:**
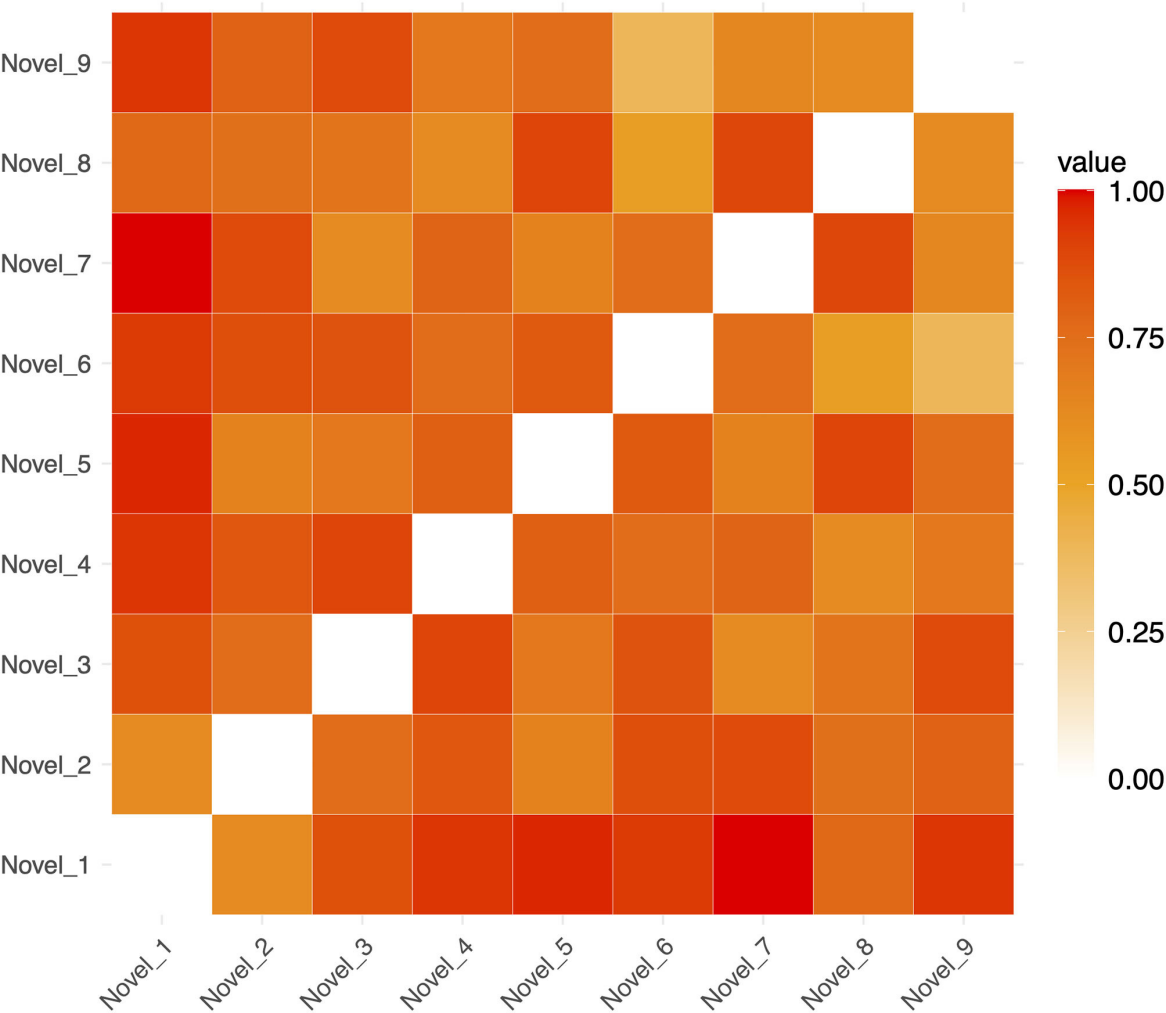
Example Representational Dissimilarity Matrix for novel sequences in a single run (for ease of visualization). The dissimilarity between any pair of sequences is indicated by the color of in the cells of the matrix.

**Figure 5:**
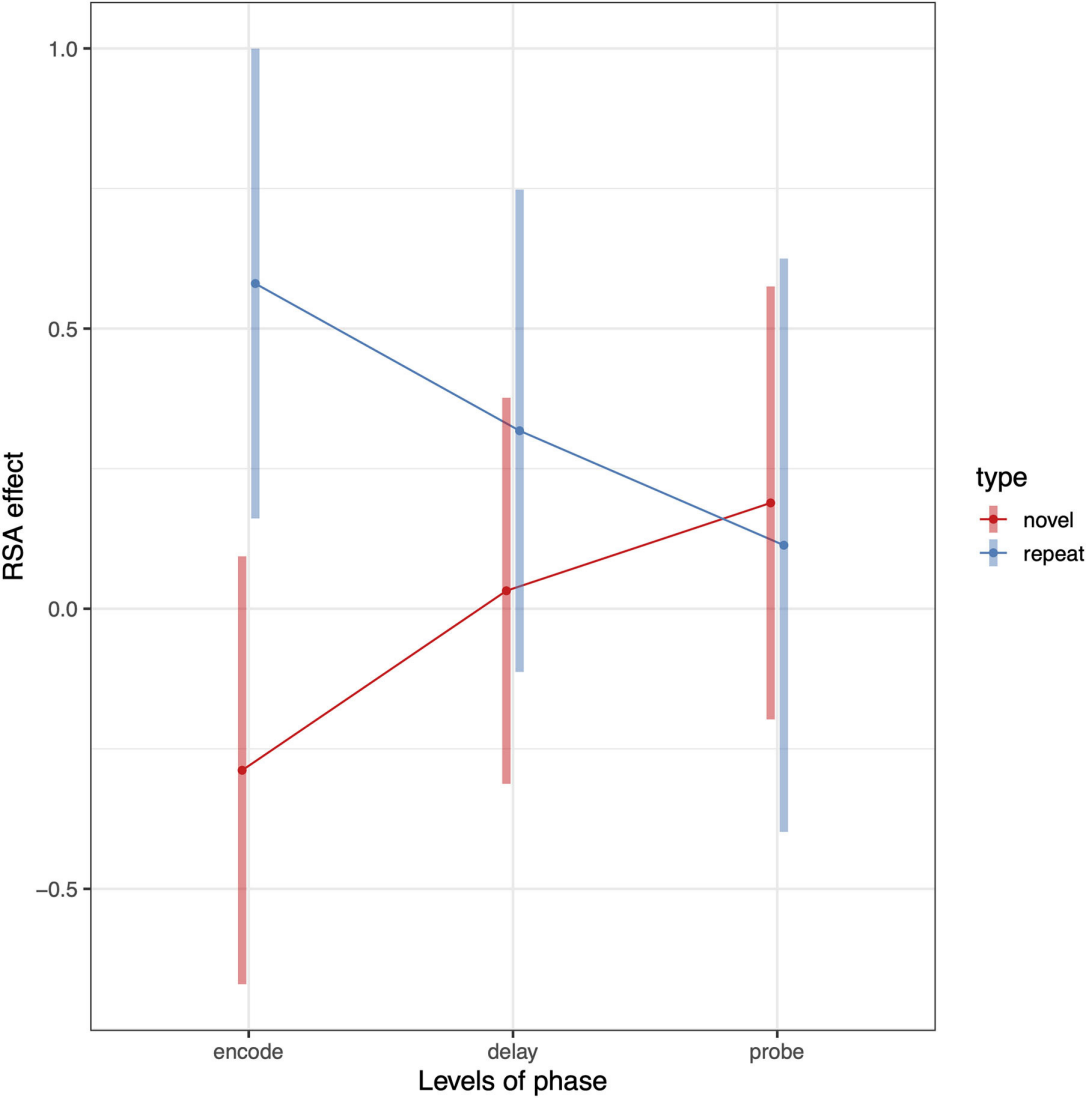
Pattern of RSA effects in Spt/PSL for repeated sequence and novel sequence RSA analyses. Error bars are 95% confidence intervals.

To test whether Spt patterns carry sequence specific information we computed a 3 (phase) × 2 (sequence type) × 2 (hemisphere) linear mixed effects model where the dependent variable was the association between the relevant RDM [e.g., novel or repeated; example matrices shown in Figure 3 (repeated sequences) and Figure 4 (novel sequences)] and the pattern activity matrix for each phase and hemisphere. Random effects were including for the subject intercept and the slope terms for phase (encoding, delay, probe) and sequence type (novel or repeated). Because hemisphere was not a significant factor (no main effect or interactions), we removed it from the model and recomputed a simpler 3 (phase) × 2 (sequence type) mixed effects model. Consistent with a specific effect of sequence repetition, there was a significant interaction between phase and type [*F*_(2,203)_ = 4.67, *p* = 0.0104). As can be seen in the plot of estimated marginal effects shown in Figure 5, the difference between the RSA effects for repeated and novel sequences is largest during the encoding phase, intermediate for the delay phase, and near zero for the probe phase.

The above analysis established that activity patterns systematically differ between repeated and novel trials and that the effect is phase-dependent. We can also test whether the RSA effects for repeated sequences are >0 by removing the novel conditions from the model. To do this we conduct a second linear mixed model analyses including only repeat trials and compute contrasts against zero for each level of the phase factor. The RSA effect for the encoding phase was significantly >0 (*t* = 3.03, *df* = 22, *p* = 0.006); the delay phase showed a trend level effect (*t* = 1.53, *df* = 22, *p* = 0.14); and there was no effect for the probe condition (*t* = 0.43, *df* = 22, *p* = 0.66). This general pattern is also evident from in the plot shown (blue line) in Figure 5.

**Figure 6:**
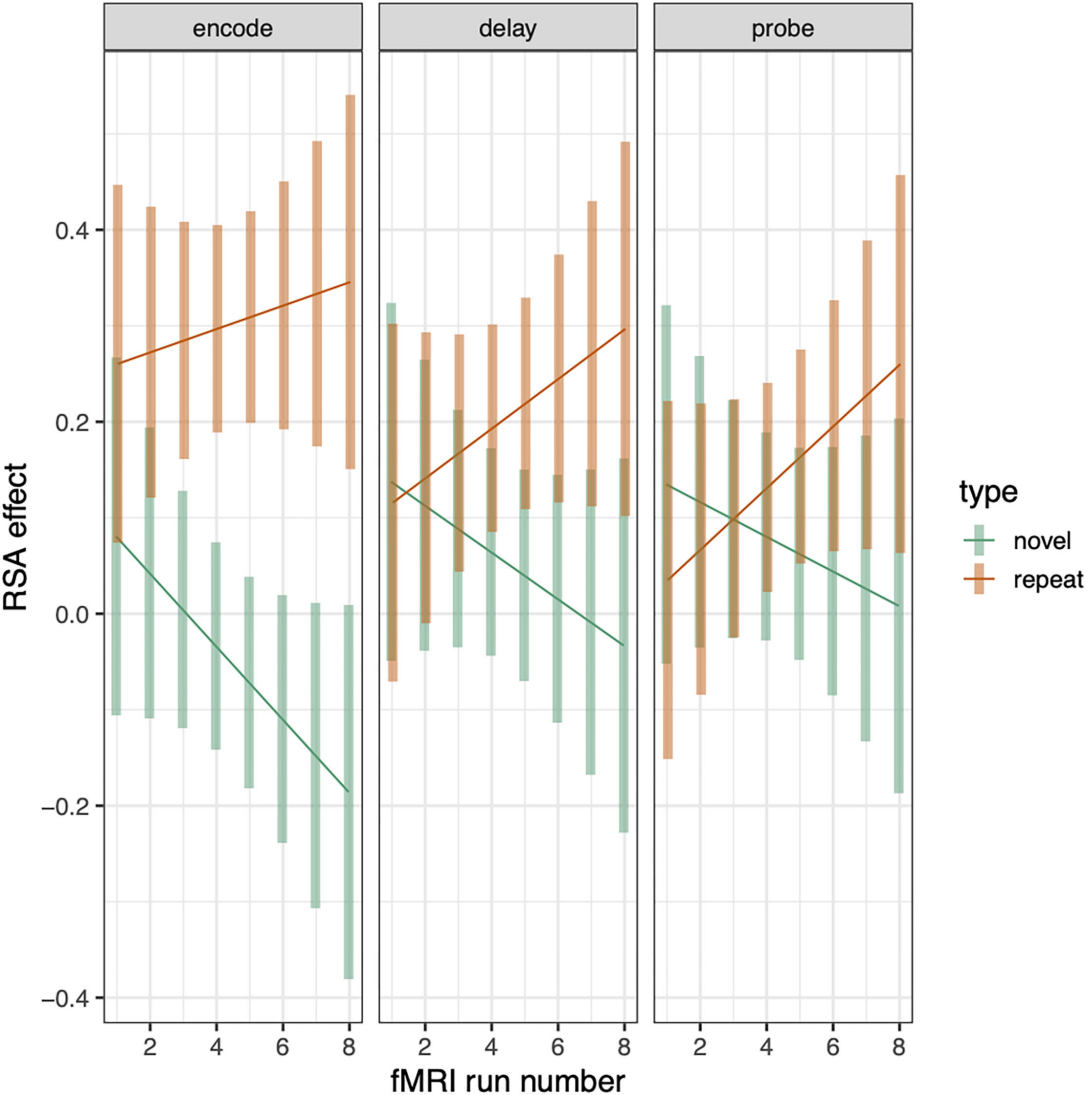
RSA effect in Spt/PSL split by sequence type and plotted across run. Error bars are 95% confidence intervals.

The above analyses examine RSA sequence similarity effects for repeated and novel trials and establish that the relationship between tone-sequence similarity and trialwise pattern similarity within PSL is greater for repeated than novel trials. This analysis pools over all trials and therefore does not establish whether this (repeated—novel) RSA effect increases as a function of repetition number, which would be consistent with a gradually increasing separation between the repeated sequences. To examine whether the RSA effect changes with repetition number, we recomputed the RSA effects for novel and repeated conditions separately for each scanning run (1–8). This resulted in eight RSA effects for each combination of subject, phase (encode, delay, probe), and sequence type (repeated or novel). To test for RSA increases as a function of repetition, we computed a linear-mixed effects model as before except now adding the addition “run” variable as a linear covariate, yielding a 3 (phase) × 2 (sequence type) × run (1–8) regression. This analysis revealed that the differential RSA effect (repeat—novel) increased as a function of run number (sequence type by run interaction: *t* = 2.508, *p* = 0.009). The positive t-statistic indicates that the slope of RSA effects over runs was greater for repeated than for novel trials. This trend effect did not, however, interact with phase (see Figure 6).

### Representational Similarity Analysis in Auditory-Motor Network

**Figure 7:**
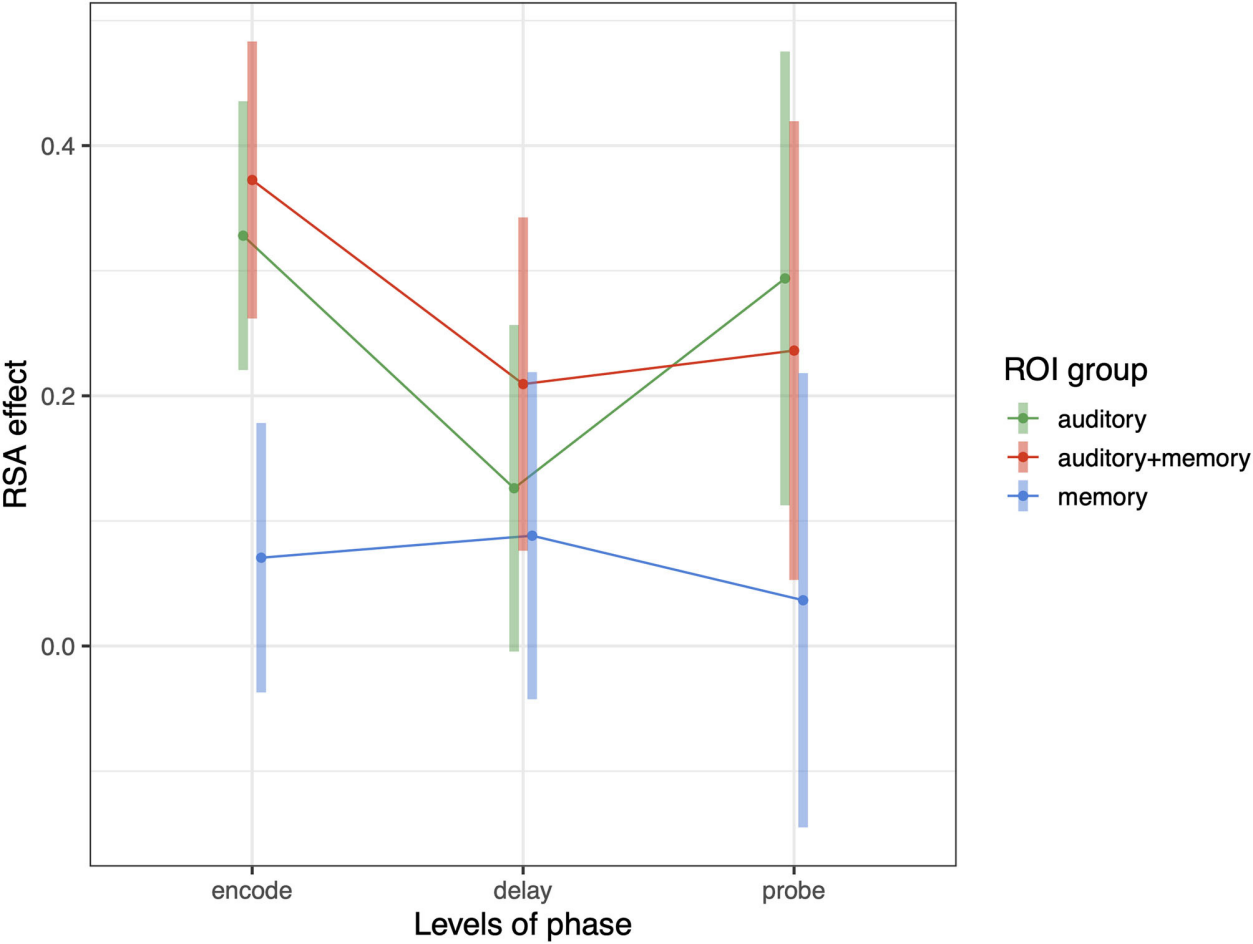
Average RSA effect in each of three ROI groups: auditory, auditory + memory, and memory. Error bars are 95% confidence intervals.

The foregoing sections show that Spt contains information about repeated tone sequences in its trial-to-trial activity patterns. Here we examine whether other regions also contain such information, and how RSA effects relate to broad univariate responses profiles in ROIs grouped in terms of their univariate response properties. In previous work examining auditory-verbal short-term memory with fMRI, we have identified sets of regions that activate during (1) auditory input (but not during memory retention), (2) auditory input *and* memory maintenance, and (3) only during memory maintenance [e.g., (13, 25)]. Here we examine whether the activity patterns associated with repeating tone sequences differ according to the groups of ROIs defined above (and depicted in Figure 2). We conducted a linear mixed effects model with RSA effect (averaged over each ROI group within subject) as the dependent variable, and with phase (encoding, delay, probe) and ROI group (auditory, auditory + memory, and memory) as the independent variables. There was also a random intercept term for subject and random slopes for ROI group and phase. We found significant effects of ROI group [*F*_(2,34.5)_ = 6.38, *p* = 0.004] and an ROI group by phase interaction [*F*_(4,7,228)_ = 3.38, *p* = 0.008); and the pattern of effects are displayed in Figure 7. Generally, the auditory and auditory + memory ROI groups showed similar patterns, where all phases were significantly above zero. In contrast, the “memory” ROI group showed effects at all phases that overlapped with zero, indicating no evidence for sequence specific activation patterns in the average effect for these ROIs (blue areas in Figure 2).

To determine whether these RSA effects change as a function of repetition, we repeated the analysis computed above for PSL in which we included “run” as an additional covariate. We performed three separate 3 (phase) × 2 (sequence type) × run (1–8) linear mixed effects models, one for each ROI group (“auditory,” “auditory + memory,” “memory”). In the auditory and auditory + memory models, we found a significant sequence type by run interaction (auditory: *t* = 3.65, *p* < 0.0004; auditory + memory: *t* = 2.165, *p* < 0.04) and in the memory model we found a trend in the same direction, Thus, generally for all ROI groups RSA effect increased as a function of run (i.e., there was a significant difference in the slopes of repeat and novel trials). There was no higher-order interaction with “phase,” i.e., the positive differential slope was similar across encoding, delay, and probe periods.

**Figure 8:**
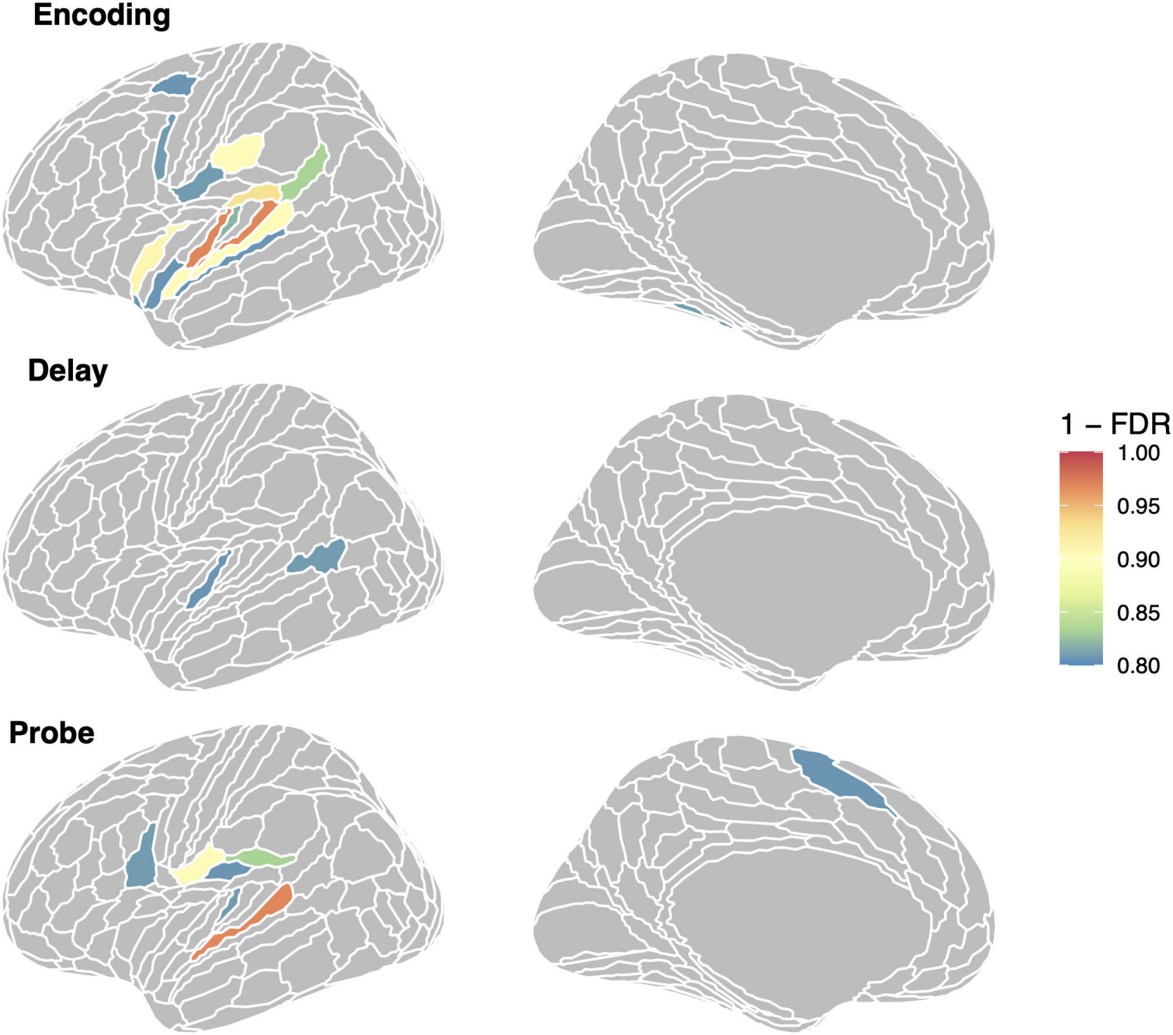
Regions showing repeated sequence RSA effects in the auditory-motor network. We show all regions with (1–FDR > 0.8); region in orange/red are significant at the FDR < 0.05 level.

To examine the specific ROIs that are driving the average effects for the auditory and auditory + memory groups reported above, we computed linear mixed model analyses with RSA effect for repeated sequences as a dependent variable and phase as an independent variable, for every ROI in the set of three groups shown in Figure 2. For each ROI (collapsing across hemisphere) and phase we then computed a contrast to test if it was significantly above zero and then corrected for multiple comparisons using FDR. In Figure 8, we show all regions with an FDR < 0.05 (orange/red) and all regions with FDR < 0.2 in cooler colors for visualization. For the encoding regions, two regions showed significant repeated sequence RSA effects, Glasser ROIs (“MBelt,” “PBelt”) in the medial and posterior regions of the auditory belt. There were no significant effects for the delay phase, and one significant effect for the probe phase in the “A4” ROI in the superior temporal gyrus (see Figure 8, bottom panel).

### Hippocampal RSA Analysis

Finally, we examined RSA effects in the hippocampus due to its known role in sequence representation and prior work that has shown sequence specific activity patterns in a version of the Hebb Repetition paradigm [e.g., (27)]. We used a mask of the hippocampus based on a probabilistic atlas defined using manual segmentation in an external group of 40 adult participants according a published protocol [OAP protocol in (56)] which we used in a previous study (57). We found significant (*p* < 0.05) RSA effects for sequence repetition in the right hippocampus for the delay period only; however, this effect did not survive multiple comparisons using FDR correction.

## Discussion

In the current study we examined whether functional-anatomical structures that are known to be involved in auditory perception and short-term maintenance (or both) also show evidence of long-term learning in a Hebb repetition paradigm with 9-item tone sequences. We were most specifically interested in examining whether Spt, a region that has repeatedly been implicated in tasks requiring the short-term maintenance of auditory-verbal and auditory-tonal sequences, operates as a pure “buffer” —wiping its contents clean from trial to trial—or whether we might detect evidence for the persistence of sequence-specific representations that spanned across trials. Indeed, our RSA analysis showed that activity patterns were more similar *within* repeated tone sequences, than they were *between* repeated tone sequences, especially during the encoding and probe phases of the task. Moreover, this effect could not be explained by tone-sequence similarity *per se*, because we did not find an overall effect of sequence-similarity among novel sequences.

In an exploratory analysis that examined the set of ROIs that showed an elevated average univariate response during (a) encoding alone, (b) during encoding and delay, and (c) delay alone, we found that the average RSA effect in the first two ROI groups (a and b) was >0 for all three trial phases, suggesting that sequence-specific information for repeated items is widely distributed across auditory-motor regions of the frontal and temporal lobes. Moreover, we found that multivoxel activity for the repeated tone sequences became more differentiated in Spt and the broader set of auditory and auditory + memory responsive ROIs, indicating that the brain's response was becoming more stable for repeated sequences as the task progressed (58–60). Prior work has shown univariate repetition suppression effects in similar lateral temporal auditory and dorsal premotor regions during a word-learning task (61) and a task involving the repetition of unfamiliar lyrical tunes [(62); see also (63)].

The current study has a direct precursor in the work of Kalm et al. (27), which used a similar approach to examine letter-sequence representations in a Hebb repetition paradigm. They showed that repeated sequence-specific representations emerged in several brain regions during letter sequence encoding, including the left hippocampus, bilateral insula, and right supramarginal gyrus. Interestingly, all of these regions fall outside the “auditory-motor” zone that we focused our multivariate analyses on. Although they found univariate repetition sequence-specific suppression effects in lateral temporal regions in the STG and STS, these regions did not show multivariate learning related sequence-specific effects. Thus, the work of Kalm et al. show that sequence-specific representations emerge *outside* the auditory-language zone, we show that learning-related representations are also evident in the same regions that process and maintain auditory-verbal information in “online” processing tasks. These results are not necessarily in conflict. In order to maximize statistical power to address particular questions about particular brain region(s), we adopted a strong *a priori* approach, focusing first on a single brain ROI (area Spt/PSL) and then “opening up” our investigations to a broader, but still limited, set of regions defined by a univariate analysis—and this set did not contain the hippocampus, supramarginal gyrus, or insula which were identified by Kalm et al. (27), Kowialiewski et al. (26) (hippocampus and insula, only).

The observed marginally significant RSA effect for repeated sequences in the right hippocampus is consistent with previous work that has demonstrated the involvement of this structure in long-term and working memory for acoustic information (64). Moreover, in view of the sequential nature of the stimuli used in the present study, this hippocampal effect aligns with studies that have implicated the hippocampus in supporting memory for the temporal order of visually experienced events [e.g., (65–69)]; and points toward a domain general role for the hippocampus in temporal order memory. More broadly, the hippocampus has been suggested to process temporal information such as order, but also duration, in support of memory in its role as a sequence processor (70, 71).

The “Hebb repetition effect” traditionally refers to an increase in the number of items recalled as a function of repetition number; however, in the current study, recognition memory performance did not increase over the experiment. Nevertheless, we did observe a repetition-related effect measured at the *neural level*, using a method that tracks multivoxel activity patterns. If a change in the brain was occurring as a function of repetition, why did we not see this effect in terms of behavioral performance? We can only speculate that our recognition memory test, which involved the difficult detection of the swapping of two tones in adjacent positions, was not sensitive to the associated neural changes that we picked up with RSA. We suspect that a tone-sequence learning task with a recall component would probably reveal significant Hebb repetition effects, but this awaits empirical examination.

In conclusion, numerous studies have shown short-term persistence of neural activity during working memory maintenance—an effect that demonstrates the existence of something like the Hebbian “activity trace” thought to operate in the service of sensory-motor control and in bridging temporal gaps that intercede between perception and action. But do these brain regions that subserve working memory maintenance act like eraser boards—holding information for a few seconds before wiping the slate clean without a trace—or do such regions keep a kind of representational inventory of the information that passes through it? Here we show that area Spt, a region often implicated in short-term memory for auditory-verbal and auditory-tonal memory, does indeed appear to maintain a record of the past, as evinced by sequence-specific multivariate pattern activity; and moreover, that this persistence of pattern information is widely distributed across auditory-motor regions in the frontal and temporal lobes.

## Data Availability Statement

The raw data supporting the conclusions of this article will be made available by the authors, without undue reservation.

## Ethics Statement

The studies involving human participants were reviewed and approved by Baycrest Research Ethic Board. The patients/participants provided their written informed consent to participate in this study.

## Author Contributions

CL analyzed data and wrote paper. AL conceived of analyses and wrote paper. BB designed study, conceived of analyses, analyzed data, and wrote paper. All authors contributed to the article and approved the submitted version.

## Funding

This work was supported by the CIHR project grant PJT152879 and NSERC Discovery award RGPIN 386631-10.

## Conflict of Interest

The authors declare that the research was conducted in the absence of any commercial or financial relationships that could be construed as a potential conflict of interest.

## Publisher's Note

All claims expressed in this article are solely those of the authors and do not necessarily represent those of their affiliated organizations, or those of the publisher, the editors and the reviewers. Any product that may be evaluated in this article, or claim that may be made by its manufacturer, is not guaranteed or endorsed by the publisher.
